# Supplementary material for: The microbiome biomarkers of pregnant women’s vaginal area predict preterm prelabor rupture in Western China
Source: Front Cell Infect Microbiol. 2024 Oct 31;14:1471027. doi: 10.3389/fcimb.2024.1471027 (PMC11560878; doi:10.3389/fcimb.2024.1471027)
Supplement: Supplementary file 1 [file DataSheet1.zip › compare_1/Community/Pieplot/species/sample/C17.species.pieplot.pdf]

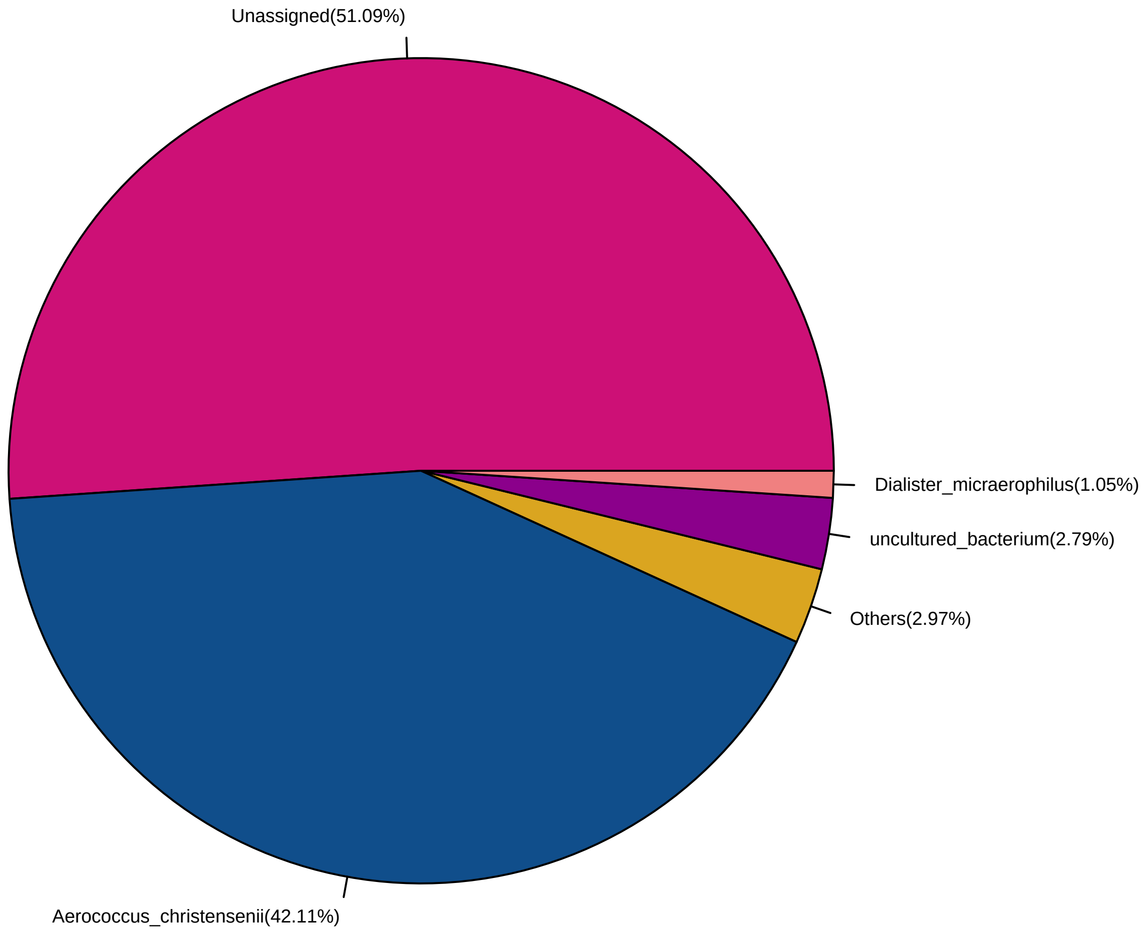
Unassigned(51.09%)
Dialister_micraerophilus(1.05%)
uncultured_bacterium(2.79%)
Others(2.97%)
Aerococcus_christensenii(42.11%)
